# Forest Management Practice Influences Bird Diversity in the Mid-Hills of Nepal

**DOI:** 10.3390/ani12192681

**Published:** 2022-10-05

**Authors:** Bijaya Neupane, Bijaya Dhami, Shristee Panthee, Alyssa B. Stewart, Thakur Silwal, Hem Bahadur Katuwal

**Affiliations:** 1 Institute of Forestry, Pokhara Campus, Tribhuvan University, Pokhara 33700, Nepal; 2 Department of Forest Sciences, Faculty of Agriculture and Forestry, University of Helsinki, 00014 Helsinki, Finland; 3 CAS Key Laboratory of Tropical Forest Ecology, Xishuangbanna Tropical Botanical Garden, Chinese Academy of Sciences, Menglun 666303, China; 4 Department of Plant Science, Faculty of Science, Mahidol University, Bangkok 10400, Thailand; 5 Center for Integrative Conservation, Xishuangbanna Tropical Botanical Garden, Chinese Academy of Sciences, Menglun 666303, China

**Keywords:** beta diversity, forest management, seasonal variation, feeding guild, bird assemblages

## Abstract

**Simple Summary:**

The conservation of biodiversity depends on the viability of management regimes. In most cases, it is well established that government-managed lands aid in the conservation of biodiversity; however, little is known regarding the conservation effectiveness of community-managed areas (e.g., community forests) in relation to government-managed areas. In the mid-hill region of Nepal, we evaluate the effectiveness of community-managed and protected forests in supporting avian diversity. We discovered greater bird diversity, richness, and abundance in the community-managed forest compared to the protected forest. In addition, the turnover of bird species was greater in the community-managed forest compared to the protected forest. Our study demonstrates that community-managed forests can have a greater diversity and abundance of bird species than government-managed protected forest.

**Abstract:**

Forest management practice plays a critical role in conserving biodiversity. However, there are few studies on how forest management practice affects bird communities. Here, we compare the effectiveness of the Panchase Protection Forest (PPF; protected forest with government administration) and the Tibrekot Community Forest (TCF; community forest with community forest users’ group administration) in hosting bird diversity in the mid-hills of Nepal. We examined 96 point count stations during summer and winter in 2019 and recorded 160 species of birds with three globally threatened vultures (red-headed vulture *Sarcogyps calvus*, slender-billed vulture *Gyps tenuirostris*, and white-rumped vulture *Gyps bengalensis*). Forest management practice, season, and elevation all influenced the richness and abundance of birds. The diversity, richness, and abundance of birds and the most common feeding guilds (insectivore, omnivore, and carnivore) were higher in TCF than in PPF; however, globally threatened species were only recorded in PPF. We also recorded a higher bird species turnover (beta diversity) in TCF than in PPF. Our study indicates that community-managed forests can also provide quality habitats similar to those of protected forests managed by the government, and provide refuge to various bird species and guilds. However, we recommend more comparative studies in other tropical and sub-tropical areas to understand how different forest management practices influence bird diversity.

## 1. Introduction

Biodiversity protection and conservation is highly dependent on the management process and intervention by managers [1,2]. Governments are often responsible for establishing or managing protected areas or forests to conserve biodiversity and enhance ecosystem services [3,4]. However, some protected areas or forests outside of protected areas are handed over to local communities for management and utilization at local levels [5]. Government-managed and community-managed protected areas are known to differ in several respects, but the impacts of these differences on biodiversity persistence are still relatively unknown. Although it is well established that government-managed areas help conserve biodiversity in most cases [6,7,8], there is meager information available about the effectiveness of community-managed areas (e.g., community forests) in conserving biodiversity [9,10] in relation to government-managed areas (see [11,12]). Identifying the value of community-managed forests in protecting biodiversity, in comparison to forests overseen by government administration, will ultimately reduce the burden on the government in terms of both the time and financial resources spent on species conservation.

The Himalayas are a hotspot of biodiversity [13]. However, due to anthropogenic disturbances, the area is facing several challenges [14]. Therefore, there is a need for sustainable forest management to conserve threatened biodiversity [15]. Birds are important indicators of forest ecosystems [16,17] and monitoring them can help to understand how forest ecosystems are changing [18]. Specifically, bird diversity and community assemblage are significantly impacted by forest management practice and intensity, as well as forest type, forest structure, and compositional heterogeneity [2,19,20,21]. However, the impact of management intervention on bird diversity and different feeding guilds (i.e., species exploiting resources in a similar manner) varies with topography (e.g., mountains versus plains), climate (e.g., tropical versus temperate areas), and seasonal changes [22,23]. Elevation gradient is another important factor in shaping bird assemblages in the Himalayas; most studies report hump-shaped patterns with the highest diversity at intermediate elevations [23,24,25], while some have found a monotonic decline with increasing elevation [26,27]. Few studies have examined the impact of forest management practice on bird community composition in South Asia, including Nepal (see [12]). In Nepal, most published studies were conducted in a single forest management practice, but few have compared how bird assemblages change across different forest management practices [11,23]. Therefore, identifying the role of forest management practice in maintaining bird diversity is necessary, as the government has continued to hand over large numbers of forest patches to the local communities in Nepal [5].

Nepal is a Himalayan country. Around 45% of the total land area of Nepal is forested [28]. The second amendment of the Forest Act 2016 broadly classified forests into two major classes: national forests and private forests [29]. National forests are further sub-categorized into government-managed forests, protected forests, community forests, collaborative forests, block forests, leasehold forests, and religious forests [29]. This study examined a protected forest and a community forest to better understand how management practice affects bird diversity (see Table 1). Community forests, which are the traditionally dominant management system in Nepal, are given to local communities to manage with active participation for the joint welfare of the local community and the forest itself [30]. In contrast, protected forests are solely administered by the government. Community forests not only contribute to the protection of native species, including threatened and endangered species, they also contribute to the livelihood of forest-dependent communities [8]. On the other hand, protected forests focus primarily on conservation benefits, which involve some wildlife management, but they provide minimal consideration to the requirements of local communities, which is in contrast to community forestry [8].

In this study, our objective was to understand the value of community-managed forests for bird composition in relation to protected forests managed by the government. We explored overall bird diversity as well as their different functional groups under two different forest-management practices in the mid-hills of Nepal. We chose the mid-hills region because this area is relatively understudied, and there are limited data on bird communities in the mid-hills compared to both higher (see [23]) and lower elevations (see [11]). Here, we selected the Panchase Protection Forest (PPF) as a protected forest managed by the government and the Tibrekot Community Forest (TCF) as a community forest managed by the community forest users’ group as a case study. We also identified the factors affecting bird richness and abundance and examined how beta diversity differs within the two forest-management practices (i.e., community versus protected). Considering the effectiveness of the government’s administration, we hypothesized higher bird diversity in protected forest (i.e., PPF) than in community forest (i.e., TCF).

## 2. Materials and Methods

### 2.1. Study Area

**Figure 1:**
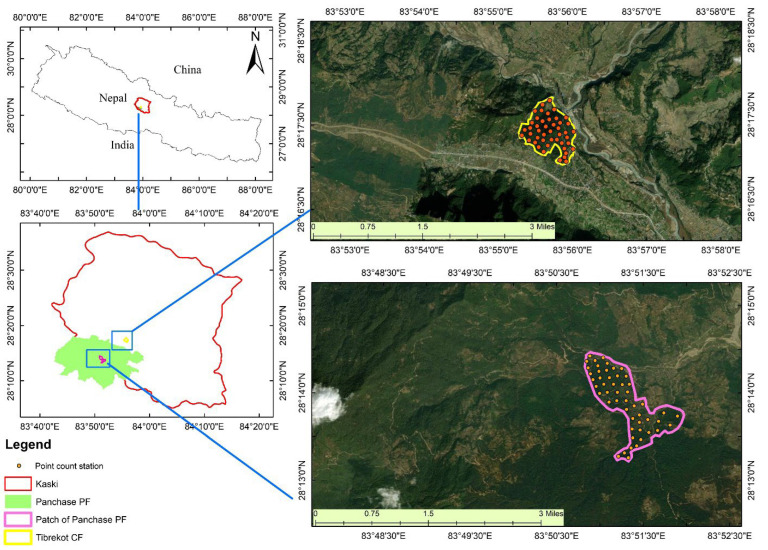
Map of the study area showing point count stations along Panchase Protection Forest (protected forest) and Tibrekot Community Forest (community forest) of Kaski district, Nepal. The protected forest is managed by the government, while the community forest is managed by the community forest users’ group.

The study was conducted in two different sites within Kaski district, Nepal: Tibrekot Community Forest (TCF) located in Hemja of Pokhara Metropolitan City ward number 25, and a patch of Panchase Protection Forest (PPF) located in Bharang of Pokhara Metropolitan City ward number 23 (Table 1; Figure 1). The total area of TCF is 120 ha and it was handed over to the local community user groups in 2000, while the total study area of PPF is 130 ha (Table 1). Both study sites are similar in biophysical characteristics, but differ in management modalities, with TCF being managed by the local people as a community forest users’ group and PPF being managed by the government. The distance between the two forests is 20 km. The elevation at both sites ranges from 900 m to 1400 m, and the dominant tree species consist of *Schima wallichii* and Castanopsis indica (Table 1).

### 2.2. Research Design and Bird Survey

We used point counts to survey birds at both sites, following existing trails and new trails, and covering the entire forest area from 900 to 1400 m [35,36]. We conducted 46 point counts in TCF and 50 point counts in the core area of PPF. The distance between two consecutive points was at least 200 m to avoid repeat counts of the same individuals. After arriving at each point, we allocated five minutes of settling time (for normal bird activity to resume), and then recorded all birds seen or heard within a 20 m radius for 10 min [37]. On each observation day, birds were surveyed from 06:30 to 10:00 and 16:30 to 18:00. The survey was conducted in the winter (January) and summer (August) of 2019, spending 15 days at each site in each season. We avoided conducting surveys on days with rain and strong winds, particularly in the summer. We used binoculars and a spotting scope to aid in species identification and took photographs of the unknown species. We used *Birds of Nepal* [38] as a field guide, and a few unidentified bird species were later verified with the help of bird experts.

### 2.3. Data Analysis

We combined all data from each forest and within each season for analysis. We classified the functional traits of species as follows: migration status (resident, winter visitor, summer visitor, and partial migrant) based on Inskipp et al. [39], conservation status (least concern, near-threatened, vulnerable, endangered, and critically endangered) based on IUCN [40], and feeding guild (insectivore: mainly eating insects, and sometimes small vertebrates; carnivore: primarily eating vertebrate prey, including carrion; omnivore: diverse and varied animal and plant diet; frugivore: mainly eating fruit and nectar with a few seeds and insects; granivore: mainly eating seeds with a few fruits and insects; piscivore: mainly eating fish with a few insects or small vertebrates; and nectarivore: mainly nectar) based on Katuwal et al. [23,41] and personal observation over the decades.

We calculated sample size-based rarefaction and extrapolation curves for all bird species from each of the two forest management types using the ‘iNEXT’ package [42,43,44]. We calculated bird diversity in each forest with Shannon’s diversity index using the ‘BiodiversityR’ package [45]. We used generalized linear models with Poisson distribution to examine the factors affecting bird richness and abundance using the ‘lem4’ package [46]. Our response variables were bird species richness and abundance and our predictor variables were forest management practice (community forest (TCF) versus protected forest (PPF)), season (winter and summer), and elevation. The Poisson models for both richness and abundance showed significant overdispersion, so we used negative binomial distribution models, which did not show overdispersion. We used ‘overdisp_fun’ function to check the dispersion of the models. We performed Tukey’s pairwise comparison to test for significant differences between factor levels using the ‘multcomp’ package [47]. We prepared the figures using predicted values from the ‘jtools’ package [48]. Finally, we also compared bird species turnover in composition (beta diversity) between the forest management types using the Jaccard dissimilarity matrix in the ‘adespatial’ package [49]. All analyses were conducted in R [50]. 

## 3. Results

Altogether, we recorded 160 bird species during the survey (Supplementary Table S1). Resident birds (n = 139) were much more common than migratory birds (n = 21; winter visitor = 13, summer visitor = 7, and one partial migrant). Around 96.25% (n = 154) of the birds were classified as least concern, while 1.8% were classified as critically endangered (n = 3; red-headed vulture *Sarcogyps calvus*, slender-billed vulture *Gyps tenuirostris*, and white-rumped vulture *Gyps bengalensis*) and another 1.8% were classified as near-threatened (n = 3; Himalayan griffon *Gyps himalayensis*, cinereous vulture *Aegypius monachus,* and mountain hawk-eagle *Nisaetus nipalensis*). We recorded three globally threatened and two near-threatened species in PPF, while one near-threatened species was observed in TCF. Sixty-one percent of the birds were insectivores (n = 98), the most common feeding guild observed, followed by omnivores (14%; n = 23), carnivores (13%; n = 21), frugivores (5%; n = 8), and granivores (4%; n = 6); nectarivores and piscivores were rarely observed.

**Figure 2:**
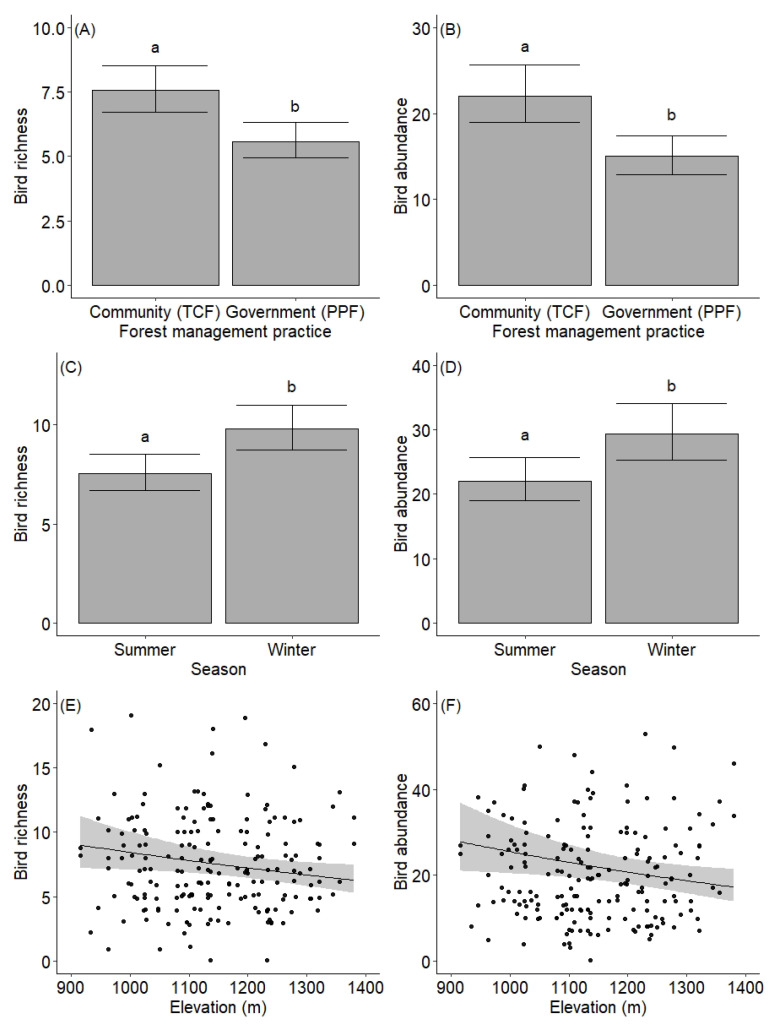
Predicted variation in species richness and abundance of birds by forest management practice (**A**,**B**), season (**C**,**D**) and elevational gradients (**E**,**F**) in the study area. The forest management practices are protected forest (Panchase Protection Forest), and community forest (Tibrekot Community Forest). The protected forest is managed by the government, while the community forest is managed by the community forest users’ group. (**A**–**D**) depict means ± 95% confidence intervals and different letters above the error bars denote significant differences between forest management practices.

**Figure 3:**
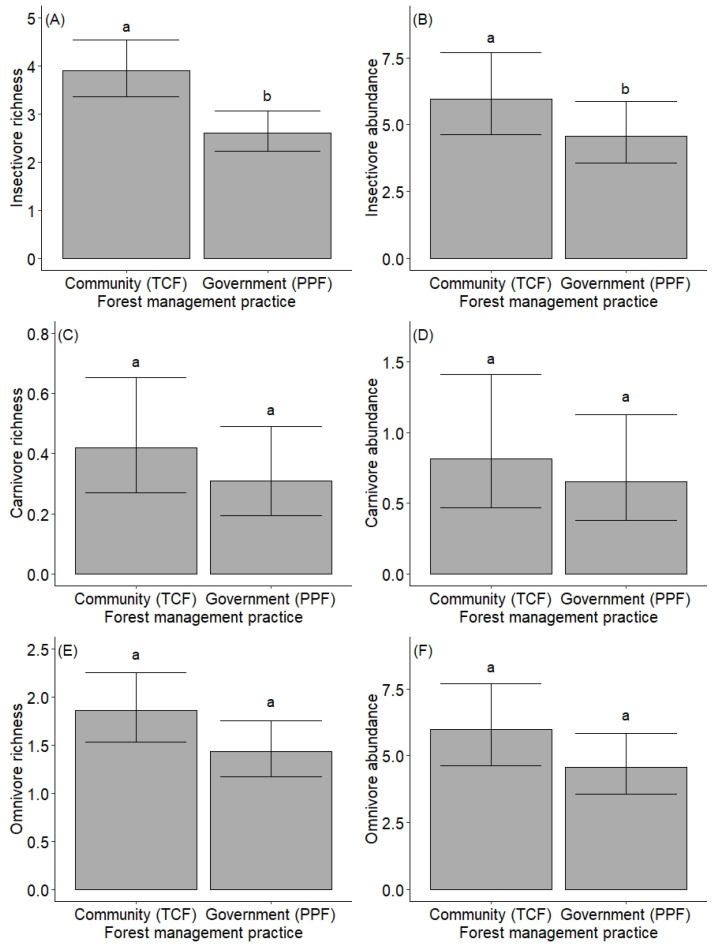
Predicted variation in bird feeding guild (**A**,**C**,**E**) richness and (**B**,**D**,**F**) abundance by forest management practice. The forest management practices are protected forest (Panchase Protection Forest) and community forest (Tibrekot Community Forest). The protected forest is managed by the government, while the community forest is managed by the community forest users’ group. Graphs depict means ± 95% confidence intervals and different letters above the error bars denote significant differences between forest management practices.

Bird species richness and abundance were highly influenced by forest management practice, season, and elevation (Table 2, Figure 2). We recorded more bird species in TCF (n = 148) than in PPF (n = 121). Pairwise comparison revealed significant differences in bird richness and abundance between forest management practices (*p* < 0.001, Figure 2A,B). Bird diversity was also higher in TCF (mean 1.82 ± 0.43 SD) than in PPF (mean 1.60 ± 0.59 SD). However, the rarefaction curves revealed that bird diversity would likely increase in both forests with greater sampling efforts (Supplementary Figure S1). More bird species were recorded in the winter (n = 155) than in the summer (n = 114; Supplementary Table S1), and pairwise comparison also showed significant differences between seasons for both bird richness and abundance (*p* < 0.001, Figure 2C,D). The richness and abundance of birds were also higher in winter than summer for both TCF and PPF. Both bird richness and abundance declined with increasing elevation (Table 2; Figure 2E,F). We also recorded higher bird species turnover (beta diversity) in TCF (72%) than in PPF (64%). Additionally, the richness and abundance of the most common feeding guilds (i.e., insectivore, carnivore, and omnivore) were also slightly higher in TCF than in PPF (Figure 3).

## 4. Discussion

Our study shows that community forests can host higher bird richness and abundance if properly managed than protected forests that are administered entirely by the government. However, some birds, including globally threatened species, appear to mainly utilize the protected forest due to extensive unlogged natural vegetation. 

Forest management practice was found to play an important role in bird species richness and abundance. The higher richness and abundance of birds in TCF might be due to the higher habitat heterogeneity in TCF than in PPF. It is possible that systematic management activities such as pruning, thinning, and cleaning may create habitat heterogeneity in TCF as it alters the vegetation composition and also induces moderate disturbance, resulting in more bird species. Moreover, TCF lies close to human settlements and the Seti River, providing suitable habitat for all kinds of urban, water-dependent, and forest-dependent birds. Berg [51] also found that habitat heterogeneity favored high avian richness and abundance. In contrast, PPF seems to support mostly forest-dependent bird species due to the abundance of unlogged natural vegetation, as Sekercioglu [52] also reported. PPF management is primarily focused on habitat improvement, species conservation, and the restoration of corridors and connectivity, which may be another reason that PPF supported more forest-dependent birds. Rayner et al. [53] concluded that physical characteristics, landscape context, and surrounding land use are important factors influencing richness and abundance, thus influencing the conservation value of protected and unprotected areas, which coincides with the results of our study, as multiple habitat types surrounded TCF. Therefore, our study shows that management activities can favorably affect the vegetation composition found in forests, and that the existing landscape around the forest, such as rivers or human settlements, influences bird composition.

Even though bird richness is higher in TCF, PPF supported rare and endangered vulture species. This pattern may be due to the availability of large trees, such as *Bombax ceiba*, which threatened vultures’ use for roosting and nesting [54,55]. However, Poudel et al. [33] observed two endangered vulture species in TCF, showing that TCF can also provide habitat for vulture species, which need long-term studies on how they utilize such trees (e.g., for scavenging, nesting, or resting).

We found the insectivore guild to be the most common feeding guild along the Himalayan forest, corresponding with previous studies [23,33]. This guild is supported by the abundant arthropods, which are also reported to be higher in the mid-hills region [56]. Although omnivores and carnivores were also relatively common, other guilds were extremely rare, possibly due to the low availability of their preferred food resources within the forest. The richness and abundance of all feeding guilds were higher in TCF than PPF. This result shows that TCF provides suitable habitats for all kinds of bird species; however, the community forest user groups also need to focus on planting tree species that provide diverse resources (e.g., fruiting trees) to support a broader variety of feeding guilds. Therefore, habitat heterogeneity favors not only overall bird diversity, but also the diversity of functional groups.

Our study found higher species richness in the winter season for both forests. The higher number of bird species in the winter (153 species) is due to the addition of the winter visitors (13 species) and a partial migrant (1 species) to the resident birds (139 species), which coincides with the results of Poudel et al. [33], a study conducted in the same TCF. The winter migrants in our study area mainly come from northern countries such as China, Mongolia, Korea, Siberia, and Central Asia in search of warmer places, whereas summer visitors come to Nepal from southern countries such as India, Sri Lanka, and Sub-Saharan Africa in search of food and breeding sites [38,39]. However, the results of Pandey et al. [24] contradict our results, as they found higher bird richness in the summer (40 species) than in the winter (32 species). The summer and winter visitors observed in our study differ from the species reported by Pandey et al. [24], despite the fact that both studies were conducted in the same study area and landscape, with only three summer migrants (blue-capped rock-thrush *Monticola cinclorhyncha*, Indian cuckoo *Cuculus micropterus*, orange-headed thrush *Geokichla citrina*) and two winter migrants (scaly thrush *Zoothera dauma*, wallcreeper Tichodroma muraria) common throughout; however, their study area encompassed a greater elevational gradient. Seasonal changes also reflect changes in temperature, precipitation, food availability, species interactions, and breeding, which can trigger bird species to migrate to avoid extreme environmental conditions [57].

Bird richness has also been shown to be influenced by elevation; however, we observed only slight declines in bird richness and abundance with increasing elevation, possibly due to the small range of elevations studied. Most studies in the Himalayan region show a hump-shaped pattern of bird richness, with the greatest diversity found at intermediate elevations [23,24,25]. However, a few studies in Nepal have also reported a monotonic decline of bird richness with increasing elevation [26,27]. As no studies have covered the entire elevational range of the Himalayas in Nepal, we recommend further studies that address the entire elevational gradients in the Nepalese Himalayas to generalize patterns of bird response towards elevation and other factors affecting their distribution.

Species turnover was relatively similar for both forest types, although slightly higher in TCF than PPF, which corresponds with the higher species richness observed in the community forest. Species turnover is known to be influenced by many factors, including habitat type [58,59,60], environmental turnover [61], species niche widths [61,62], species dispersal abilities [62], climate change, and anthropogenic activities [63]. The two study sites were once contiguous forest and thus have some similarity in terms of habitat type, so it is not surprising that they have comparable rates of species turnover. However, our two study sites differ in management practice, which may influence environmental turnover within each forest patch, which then may increase species turnover [61]. The community forest observed in this study is subject to different management activities compared to the protected forest (see Table 1), possibly contributing to higher environmental turnover within the forest patch.

## 5. Conclusions

We conclude that forests in the mid-hills of Nepal can host high bird diversity, which varies mainly by forest management practice and seasonal variation. Compared to protected forests administered by the government, community forests can have higher bird species richness and abundance across different seasons. The management techniques used in the community forest observed in this study appear not to have a negative impact on bird diversity, and may actually help to promote bird diversity. However, community forest users’ group should further manage the forest by planting native fruiting trees and safeguarding some areas from disturbance so that it can provide refugia for different kinds of bird species and functional groups, and reduce direct anthropogenic threats to the birds. Similar management activities are also suggested for the government-protected forests of our study area. Finally, we recommend further comparative studies from different tropical and sub-tropical areas to understand how birds are influenced by forest management practices.

## Figures and Tables

**Table 1:** Description of the study areas.

Characteristics	Protected Forest	Community Forest	Data Source
Name	Panchase Protection Forest	Tibrekot Community Forest	[8,31]
Area	130 ha	120 ha	[31]
Altitudinal range	900–1400 m asl	900–1400 m asl	[31]
Distance from Province’s capital (Pokhara)	18 km	12 km	
Dominant vegetation	*Schima-Castanopsis forest*	*Schima-Castanopsis forest*	[31,32,33]
Management body	Government (Division Forest Office)	Local community as community forest users’ group	[29,31,34]
Management modality	It is a part of the national forests of special environmental, scientific, or cultural importance, which aims to balance human needs through conserving biodiversity, increasing ecosystem services, and safeguarding the environment. It is divided into three zones, i.e., (a) Core area, in which intervention is prohibited and no management is applied; (b) Management zone, which is usually the area between the core area and surrounding villages and is allocated for production purposes; (c) Impact zone, which consists of villages living in the periphery of the forest where people are supported in conducting income-generating activities. The rights are not as flexible as in community forests, and the income-generating activities are from eco-tourism, payment of ecosystem services, etc.	It is a part of the national forest handed over to the local community forest users’ group living around the area to prepare a work plan with permission from the Division Forest Office under the Forest Act 1993. The main goal of the community forest is to develop, conserve, use, and manage the forest as well as sell and distribute forest products (e.g., timber every year from designed blocks) independently by fixing their prices according to the work plan, which shows high human intervention in forest areas. Additionally, different tending activities are performed such as cleaning, pruning, thinning, and felling regularly, with the right to introduce forest-based enterprises through approval from the Division Forest Office.	[8,29,34]

**Table 2:** Summary of generalized linear model results showing the factors affecting bird species richness and abundance in the mid-hills of Nepal. We used negative binomial distributions due to overdispersion in the Poisson family. The forest management practices included protected forest (Panchase Protection Forest (PPF)) and community forest (Tibrekot Community Forest (TCF)). The protected forest is managed by the government, while the community forest is managed by the community forest users’ group. The seasons included winter and summer.

Parameters	Estimate	Std. Error	z Value	Pr (>|z|)
**Richness**				
Intercept	2.90	0.42	6.84	<0.001
Forest management practice: Protected (PPF)	−0.30	0.07	−4.01	<0.001
Season: Winter	0.25	0.06	3.92	<0.001
Elevation	−0.0007	0.0003	−2.19	0.028
**Abundance**				
Intercept	4.26	0.53	7.98	<0.001
Forest management practice: Protected (PPF)	−0.38	0.09	−4.03	<0.001
Season: Winter	0.28	0.08	3.44	<0.001
Elevation	−0.001	0.0004	−2.32	0.020

## Data Availability

The data will be provided upon request by the corresponding author.

## Supplementary Materials

The following supporting information can be downloaded at: https://www.mdpi.com/article/10.3390/ani12192681/s1, Table S1: Bird species recorded during the survey in Panchase Protection Forest (protected forest) and Tibrekot Community Forest (community forest); Figure S1: Sample-size-based rarefaction (solid lines) and extrapolation sampling curves with 95% confidence intervals (shaded) representing bird communities in Panchase Protection Forest (protected forest) and Tibrekot Community Forest (community forest).

## References

[1] Black S.A., Groombridge J.J., Jones C.G. (2013). Using Better Management Thinking to Improve Conservation Effectiveness. ISRN Biodivers..

[2] Lešo P., Kropil R., Kajtoch Ł. (2019). Effects of Forest Management on Bird Assemblages in Oak-Dominated Stands of the Western Carpathians—Refuges for Rare Species. For. Ecol. Manag..

[3] Lopoukhine N., Crawhall N., Dudley N., Figgis P., Karibuhoye C., Laffoley D., Miranda Londoño J., MacKinnon K., Sandwith T. (2012). Protected Areas: Providing Natural Solutions to 21st Century Challenges. Sapiens.

[4] Watson J.E.M., Dudley N., Segan D.B., Hockings M. (2014). The Performance and Potential of Protected Areas. Nature.

[5] Pandey H.P., Pokhrel N.P. (2021). Formation Trend Analysis and Gender Inclusion in Community Forests of Nepal. Trees For. People.

[6] Cazalis V., Princé K., Mihoub J.B., Kelly J., Butchart S.H.M., Rodrigues A.S.L. (2020). Effectiveness of Protected Areas in Conserving Tropical Forest Birds. Nat. Commun..

[7] Dudley N., Phillips A., Amend T., Brown J., Stolton S. (2016). Evidence for Biodiversity Conservation in Protected Landscapes. Land.

[8] Shrestha T.K., Aryal A., Rai R.K., Lamsal R.P., Koirala S., Jnawali D., Kafle R., Bhandari B.P., Raubenheimer D. (2014). Balancing Wildlife and Human Needs: The Protected Forest Approach in Nepal. Nat. Areas J..

[9] Sayer J., Margules C. (2017). Biodiversity in Locally Managed Lands. Land.

[10] Boedhihartono A.K. (2017). Can Community Forests Be Compatible with Biodiversity Conservation in Indonesia?. Land.

[11] Dahal B.R., McAlpine C.A., Maron M. (2014). Bird Conservation Values of Off-Reserve Forests in Lowland Nepal. For. Ecol. Manag..

[12] Velho N., Sreekar R., Laurance W.F. (2016). Terrestrial Species in Protected Areas and Community-Managed Lands in Arunachal Pradesh, Northeast India. Land.

[13] Xu J., Badola R., Chettri N., Chaudhary R.P., Zomer R., Pokhrel B., Hussain S.A., Pradhan S., Pradhan R., Wester P., Mishra A., Mukherji A., Shrestha A.B. (2019). Sustaining Biodiversity and Ecosystem Services in the Hindu Kush Himalaya. The Hindu Kush Himalaya Assessment: Mountains, Climage Change, Sustainability and People.

[14] Kattel G.R. (2022). Climate Warming in the Himalayas Threatens Biodiversity, Ecosystem Functioning and Ecosystem Services in the 21st Century: Is There a Better Solution?. Biodivers. Conserv..

[15] Imai N., Samejima H., Langner A., Ong R.C., Kita S., Titin J., Chung A.Y.C., Lagan P., Lee Y.F., Kitayama K. (2009). Co-Benefits of Sustainable Forest Management in Biodiversity Conservation and Carbon Sequestration. PLoS ONE.

[16] Stratford J.A., Sekercioglu C.H. (2015). Birds in Forest Ecosystems. Handbook of Forest Ecology.

[17] Machar I., Šimek P., Schlossárek M., Pechanec V., Petrovič F., Brus J., Špinlerová Z., Seják J. (2022). Comparison of Bird Diversity between Temperate Floodplain Forests and Urban Parks. Urban For. Urban Green..

[18] Ramírez-Soto A., Rodríguez-Mesa R., Villa-Bonilla B., Sheseña-Hernández I., Inzunza E.R. (2018). Using Birds to Assess and Track Forest Restoration. Trop. Conserv. Sci..

[19] Perry R.W., Jenkins J.M.A., Thill R.E., Thompson F.R. (2018). Long-Term Effects of Different Forest Regeneration Methods on Mature Forest Birds. For. Ecol. Manag..

[20] Bergner A., Avci M., Eryiğit H., Jansson N., Niklasson M., Westerberg L., Milberg P. (2015). Influences of Forest Type and Habitat Structure on Bird Assemblages of Oak (*Quercus* Spp.) and Pine (*Pinus* Spp.) Stands in Southwestern Turkey. For. Ecol. Manag..

[21] Leitão P.J., Toraño Caicoya A., Dahlkamp A., Guderjan L., Griesser M., Haverkamp P.J., Nordén J., Snäll T., Schröder B. (2022). Impacts of Forest Management on Forest Bird Occurrence Patterns—A Case Study in Central Europe. Front. For. Glob. Change.

[22] Girma Z., Mamo Y., Mengesha G., Verma A., Asfaw T. (2017). Seasonal Abundance and Habitat Use of Bird Species in and around Wondo Genet Forest, South-Central Ethiopia. Ecol. Evol..

[23] Katuwal H.B., Basnet K., Khanal B., Devkota S., Rai S.K., Gajurel J.P., Scheidegger C., Nobis M.P. (2016). Seasonal Changes in Bird Species and Feeding Guilds along Elevational Gradients of the Central Himalayas, Nepal. PLoS ONE.

[24] Pandey N., Khanal L., Chalise M.K. (2020). Correlates of Avifaunal Diversity along the Elevational Gradient of Mardi Himal in Annapurna Conservation Area, Central Nepal. Avian Res..

[25] Ding Z., Hu H., Cadotte M.W., Liang J., Hu Y., Si X. (2021). Elevational Patterns of Bird Functional and Phylogenetic Structure in the Central Himalaya. Ecography.

[26] Ghimire A., Rokaya M.B., Timsina B., Bílá K., Shrestha U.B., Chalise M.K., Kindlmann P. (2021). Diversity of Birds Recorded at Different Altitudes in Central Nepal Himalayas. Ecol. Indic..

[27] Neupane J., Khanal L., Gyawali B., Chalise M.K. (2020). Elevational Pattern and Seasonality of Avian Diversity in Kaligandaki River Basin, Central Himalaya. J. Threat. Taxa.

[28] MFE (2019). National Level Forests and Land Cover Analysis of Nepal Using Google Earth Images.

[29] GoN (2019). The Forests Act, 2019.

[30] Pathak B.R., Yi X., Bohara R. (2017). Community Based Forestry in Nepal: Status, Issues and Lessons Learned. Int. J. Sci..

[31] Neupane B., Dhami B., Bista S., Sadadev B.M., Regmi S., Shrestha S., Shrestha B., Traxmandlová I., Varachova S., Kindlmann P. (2022). Ecological Factors Determining Barking Deer Distribution and Habitat Use in the Mid-Hills of Nepal. Front. Ecol. Evol..

[32] Bijaya G.C.D., Cheng S., Gao Q., Xu Z., Wang L., Jyoti B., Liu X., Gao L., Cao X. (2015). Can Community Forestry Play a Major Role in the Socio-Economic Enhancement of Poor Users in Nepal?. Bulg. J. Agric. Sci..

[33] Poudel B., Neupane B., Joshi R., Silwal T., Raut N., Thanet D.R. (2021). Factors Affecting the Species Richness and Composition of Bird Species in a Community Managed Forest of Nepal. J. Threat. Taxa.

[34] Gautam A.P., Bhujel K.B., Chhetri R. (2017). Political Economy of Forest Tenure Reform Implementation in Nepal: The Case of Protected Forests. J. For. Livelihood.

[35] Ralph C.J., Droege S., Sauer J.R. (1995). Managing and Monitoring Birds Using Point Counts: Standards and Applications. Monitoring Bird Populations by Point Counts.

[36] Siegel R. (2009). Methods for Monitoring Landbirds: A Review Commissioned by Seattle City Light’s Wildlife Research Advisory Committee (2000).

[37] Hostetler M., Main M.B. (2001). Florida Monitoring Program: Point Count Method to Survey Birds.

[38] Grimmett R., Inskipp C., Inskipp T., Baral H.S. (2016). Birds of Nepal (Revised Edition).

[39] Inskipp C., Baral H.S., Phuyal S., Bhatt T., Khatiwada M., Inskipp T., Khatiwada A.P., Gurung S., Singh P., Murray L. (2016). The Status of Nepal’s Birds: The National Red List Series.

[40] IUCN (2021). The IUCN Red List of Threatened Species. Version 2020-3. https://www.iucnredlist.org.

[41] Katuwal H.B., Rai J., Tomlinson K., Rimal B., Sharma H.P., Baral H.S., Hughes A.C., Quan R.-C. (2022). Seasonal Variation and Crop Diversity Shape the Composition of Bird Communities in Agricultural Landscapes in Nepal. Agric. Ecosyst. Environ..

[42] Hsieh T.C., Ma K.H., Chao A., INEXT: INterpolation and EXTrapolation for Species Diversity. R Package Version 2.0 (2020). http://chao.stat.nthu.edu.tw/wordpress/software-download/.

[43] Tiwari G., Pandey P., Kaul R., Lee H., Singh R. (2022). Time-of-Day Bias in Diurnal Raptors in Arid Region of Rajasthan. Acta Ecol. Sin..

[44] Tiwari G., Pandey P., Kaul R., Lee H., Singh R. (2021). Comparison of Point and Roadside Transect Methods to Evaluate the Abundance and Richness of Diurnal Raptors in the Arid Region of Rajasthan. PLoS ONE.

[45] Kindt R., Coe R. (2005). Tree Diversity Analysis. A Manual and Software for Common Statistical Methods for Ecological and Biodiversity Studies.

[46] Bates D., Maechler M., Bolker B., Walker S. (2015). Fitting Linear Mixed-Effects Models Using Lme4. J. Stat. Soft..

[47] Hothorn T., Bretz F., Westfall P. (2008). Simultaneous Inference in General Parametric Models. Biom. J..

[48] Long J.A. (2022). Jtools: Analysis and Presentation of Social Scientific Data. R Package Version 2.2.0. https://cran.r-project.org./package=jtools>.

[49] Dray S., Bauman D., Blanchet G., Borcard D., Clappe S., Guenard G., Jombart T., Larocque G., Legendre P., Madi N. (2022). Adespatial: Multivariate Multiscale Spatial Analysis. R Package Version 0.3-18. https://CRAN.R-project.org/package=adspatial.

[50] R Core Team (2019). R: A Language and Environment for Statistical Computing; R Foundation for Statistical Computing: Vienna, Austria. http://www.R-project.org.

[51] Berg Å. (1997). Diversity and Abundance of Birds in Relation to Forest Fragmentation, Habitat Quality and Heterogeneity. Bird Study.

[52] Sekercioglu C.H. (2002). Effects of Forestry Practices on Vegetation Structure and Bird Community of Kibale National Park, Uganda. Biol. Conserv..

[53] Rayner L., Lindenmayer D.B., Wood J.T., Gibbons P., Manning A.D. (2014). Are Protected Areas Maintaining Bird Diversity?. Ecography.

[54] Ghimire B., Acharya R., Sivakumar K., Biswas S., Dorji C. (2019). Nesting Characteristics and Habitat Preferences of Critically Endangered White-Rumped Vulture Gyps Bengalensis in Rampur IBA, Palpa. Vulture Bul..

[55] Majgaonkar I., Bowden C.G.R., Quader S. (2018). Nesting Success and Nest-Site Selection of White-Rumped Vultures (Gyps Bengalensis) in Western Maharashtra, India. J. Raptor Res..

[56] Price T.D., Hooper D.M., Buchanan C.D., Johansson U.S., Tietze D.T., Alström P., Olsson U., Ghosh-Harihar M., Ishtiaq F., Gupta S.K. (2014). Niche Filling Slows the Diversification of Himalayan Songbirds. Nature.

[57] Amani M., Salehi B., Mahdavi S., Brisco B. (2018). Spectral Analysis of Wetlands Using Multi-Source Optical Satellite Imagery. ISPRS J. Photogramm. Remote Sens..

[58] Karp D.S., Rominger A.J., Zook J., Ranganathan J., Ehrlick P.R., Daily G.C. (2012). Intensive Agriculture Erodes β-Diversity at Large Scales. Ecol. Let..

[59] De Castro Solar R.R., Barlow J., Ferreira J., Berenguer E., Lees A.C., Thomson J.R., Louzada J., Maués M., Moura N.G., Oliveira V.H.F. (2015). How Pervasive Is Biotic Homogenization in Human-Modified Tropical Forest Landscapes?. Ecol. Let..

[60] Sreekar R., Corlett R.T., Dayananda S., Goodale U.M., Kilpatrick A., Kotagama S.W., Koh L.P., Goodale E. (2017). Horizontal and Vertical Species Turnover in Tropical Birds in Habitats with Differing Land Use. Biol. Let..

[61] Buckley L.B., Jetz W. (2008). Linking Global Turnover of Species and Environments. Proc. Natl. Acad. Sci. USA.

[62] Qian H., Ricklefs R.E. (2012). Disentangling the Effects of Geographic Distance and Environmental Dissimilarity on Global Patterns of Species Turnover. Glob. Ecol. Biogeog..

[63] Virkkala R., Lehikoinen A. (2017). Birds on the Move in the Face of Climate Change: High Species Turnover in Northern Europe. Ecol. Evol..

