# Supplementary material for: Forest Management Practice Influences Bird Diversity in the Mid-Hills of Nepal
Source: Animals (Basel). 2022 Oct 5;12(19):2681. doi: 10.3390/ani12192681 (PMC9559466; doi:10.3390/ani12192681)
Supplement: Supplementary file 1 [file animals-12-02681-s001.zip › animals-1904108-supplementary.pdf]

Supplementary Files

Table S1. Bird species recorded during the survey in Panchase Protection Forest (protected forest) and Tibrekot Community Forest (community forest).

SN	Common Name	Scientific Name	Feeding guild	Migration	IUCN Status
1	Ashy Drongo	*Dicrurus leucophaeus*	Insectivore	Resident	LC
2	Ashy Bulbul	*Hemixos flavala*	Omnivore	Resident	LC
3	Ashy-throated Warbler	*Phylloscopus maculipennis*	Insectivore	Resident	LC
4	Asian Barred Owlet	*Glaucidium cuculoides*	Carnivore	Resident	LC
5	Asian Plain Martin	*Riparia chinensis*	Insectivore	Resident	LC
6	Barred Cuckoo-dove	*Macropygia unchall*	Granivore	Resident	LC
7	Bar-winged Flycatcher-shrike	*Hemipus picatus*	Insectivore	Resident	LC
8	Black Bulbul	*Hypsipetes leucocephalus*	Insectivore	Resident	LC
9	Black Drongo	*Dicrurus macrocercus*	Insectivore	Resident	LC
10	Black Francolin	*Francolinus francolinus*	Omnivore	Resident	LC
11	Black Kite	*Milvus migrans*	Carnivore	Resident	LC
12	Black-throated Sunbird	*Aethopyga saturata*	Nectarivore	Resident	LC
13	Black-chinned Babbler	*Cyanoderma pyrrhops*	Omnivore	Resident	LC
14	Black-lored Tit	*Machlolophus xanthogenys*	Insectivore	Resident	LC

15	Black-throated Tit	*Aegithalos concinnus*	Insectivore	Resident	LC
16	Black-throated Thrush	*Turdus atrogularis*	Insectivore	Winter visitor	LC
17	Blue Whistling-thrush	*Myophonus caeruleus*	Insectivore	Resident	LC
18	Black-winged Cuckooshrike	*Lalage melaschistos*	Insectivore	Resident	LC
19	Blue-bearded Bee-eater	*Nyctyornis athertoni*	Insectivore	Summer visitor	LC
20	Blue-capped Rock-thrush	*Monticola cinclorhyncha*	Insectivore	Summer visitor	LC
21	Blue-winged Minla	*Siva cyanouroptera*	Insectivore	Resident	LC
22	Blue-throated Barbet	*Psilopogon asiaticus*	Frugivore	Resident	LC
23	Blue-throated Blue-flycatcher	*Cyornis rubeculoides*	Insectivore	Partial migrant	LC
24	Black-headed Jay	*Garrulus lanceolatus*	Omnivore	Resident	LC
25	Bronzed Drongo	*Dicrurus aeneus*	Insectivore	Resident	LC
26	Buff-barred warbler	*Phylloscopus pulcher*	Insectivore	Resident	LC
27	Cattle Egret	*Bubulcus ibis*	Insectivore	Resident	LC
28	Chestnut-bellied nuthatch	*Sitta cinnamoventris*	Insectivore	Resident	LC
29	Cinereous Tit	*Parus major*	Insectivore	Resident	LC
30	Cinereous Vulture	*Aegypius monachus*	Carnivore	Winter visitor	NT
31	Collared Owlet	*Glaucidium brodiei*	Carnivore	Resident	LC
32	Collared Scops-owl	*Otus lettia*	Carnivore	Resident	LC

33	Common Barn-owl	*Tyto alba*	Carnivore	Resident	LC
34	Common Green Magpie	*Cissa chinensis*	Carnivore	Resident	LC
35	Common Hawk-cuckoo	*Hierococcyx varius*	Insectivore	Resident	LC
36	Common Hoopoe	*Upupa epops*	Insectivore	Resident	LC
37	Common Kestrel	*Falco tinnunculus*	Carnivore	Resident	LC
38	Common Myna	*Acridotheres tristis*	Omnivore	Resident	LC
39	Common Tailorbird	*Orthotomus sutorius*	Insectivore	Resident	LC
40	Crested Kingfisher	*Megaceryle lugubris*	Piscivore	Resident	LC
41	Coppersmith Barbet	*Psilopogon haemacephalus*	Frugivore	Resident	LC
42	Crested Serpent-eagle	*Spilornis cheela*	Carnivore	Resident	LC
43	Chestnut-headed Tesia	*Cettia castaneocoronata*	Insectivore	Resident	LC
44	Chestnut-bellied Rock-thrush	*Monticola rufiventris*	Insectivore	Resident	LC
45	Crimson Sunbird	*Aethopyga siparaja*	Nectarivore	Resident	LC
46	Grey-capped Emerald dove	*Chalcophaps indica*	Omnivore	Resident	LC
47	Eurasian Wryneck	*Jynx torquilla*	Insectivore	Winter visitor	LC
48	Eurasian tree sparrow	*Passer montanus*	Granivore	Resident	LC
49	Fulvous-breasted Woodpecker	*Dendrocopos macei*	Insectivore	Resident	LC
50	Golden-throated Barbet	*Psilopogon franklinii*	Frugivore	Resident	LC
51	Great Barbet	*Psilopogon virens*	Frugivore	Resident	LC
52	Greater Coucal	*Centropus sinensis*	Omnivore	Resident	LC

53	Greater Flameback	*Chrysocolaptes guttacristatus*	Insectivore	Resident	LC
54	Greater Yellownape	*Chrysophlegma flavinucha*	Insectivore	Resident	LC
55	Green shrike-babbler	*Pteruthius xanthochlorus*	Insectivore	Resident	LC
56	Green-backed Tit	*Parus monticolus*	Insectivore	Resident	LC
57	Green-billed Malkoha	*Phaenicophaeus tristis*	Insectivore	Resident	LC
58	Green-tailed Sunbird	*Aethopyga nipalensis*	Nectarivore	Resident	LC
59	Greenish Warbler	*Phylloscopus trochiloides*	Insectivore	Winter visitor	LC
60	Grey-bellied cuckoo	*Cacomantis passerinus*	Insectivore	Summer visitor	LC
61	Grey Bushchat	*Saxicola ferreus*	Insectivore	Resident	LC
62	Grey Nightjar	*Caprimulgus jotaka*	Insectivore	Resident	LC
63	Grey-bellied Tesia	*Tesia cyaniventer*	Insectivore	Resident	LC
64	Grey Wagtail	*Motacilla cinerea*	Insectivore	Resident	LC
65	Grey Treepie	*Dendrocitta formosae*	Omnivore	Resident	LC
66	Grey-backed Shrike	*Lanius tephronotus*	Insectivore	Resident	LC
67	Grey-headed Canary-flycatcher	*Culicicapa ceylonensis*	Insectivore	Resident	LC
68	Grey-hooded Warbler	*Phylloscopus xanthoschistos*	Insectivore	Resident	LC
69	Grey-headed Woodpecker	*Picus canus*	Insectivore	Resident	LC

70	Grey-throated Babbler	*Stachyris nigriceps*	Insectivore	Resident	LC
71	Hair-crested Drongro	*Dicrurus hottentottus*	Insectivore	Resident	LC
72	Hill Partridge	*Arborophila torqueola*	Omnivore	Resident	LC
73	Himalayan Bulbul	*Pycnonotus leucogenys*	Omnivore	Resident	LC
74	Himalayan Griffon	*Gyps himalayensis*	Carnivore	Resident	NT
75	Hodgson's Treecreeper	*Certhia hodgsoni*	Insectivore	Resident	LC
76	House Crow	*Corvus splendens*	Omnivore	Resident	LC
77	House Sparrow	*Passer domesticus*	Omnivore	Resident	LC
78	Hume's Leaf-warbler	*Phylloscopus humei*	Insectivore	Resident	LC
79	Indian Cuckoo	*Cuculus micropterus*	Insectivore	Summer visitor	LC
80	Indian Pond-heron	*Ardeola grayii*	Carnivore	Resident	LC
81	Indian Golden Oriole	*Oriolus kundoo*	Insectivore	Summer visitor	LC
82	Jungle Myna	*Acridotheres fuscus*	Omnivore	Resident	LC
83	Kalij Pheasant	*Lophura leucomelanos*	Omnivore	Resident	LC
84	Large Cuckooshrike	*Coracina macei*	Insectivore	Resident	LC
85	Large-billed Crow	*Corvus macrorhynchos*	Omnivore	Resident	LC
86	Large Hawk-cuckoo	*Hierococcyx sparverioides*	Insectivore	Summer visitor	LC
87	Lesser racquet-tailed Drongo	*Dicrurus remifer*	Insectivore	Resident	LC
88	Lesser Yellownape	*Picus chlorol*	Insectivore	Resident	LC
89	Little Egret	*Egretta garzetta*	Carnivore	Resident	LC

90	Long-tailed Broadbill	*Psarisomus dalhousiae*	Insectivore	Resident	LC
91	Long-tailed Minivet	*Pericrocotus ethologus*	Insectivore	Resident	LC
92	Long-tailed Shrike	*Lanius schach*	Insectivore	Resident	LC
93	Lemon-rumped Leaf-warbler	*Phylloscopus chloronotus*	Insectivore	Resident	LC
94	Maroon Oriole	*Oriolus traillii*	Omnivore	Resident	LC
95	Mountain Bulbul	*Ixos mcclellandii*	Omnivore	Resident	LC
96	Mountain Hawk-eagle	*Nisaetus nipalensis*	Carnivore	Resident	NT
97	Northern Wren	*Troglodytes troglodytes*	Insectivore	Resident	LC
98	Mountain Scops-owl	*Otus spilocephalus*	Carnivore	Resident	LC
99	Olive-backed Pipit	*Anthus hodgsoni*	Insectivore	Winter visitor	LC
100	Orange-bellied Leafbird	*Chloropsis hardwickii*	Omnivore	Resident	LC
101	Yellow-bellied Fairy-fantail	*Chelidorhynx hypoxanthus*	Insectivore	Resident	LC
102	Orange-headed Thrush	*Geokichla citrina*	Insectivore	Summer visitor	LC
103	Oriental Magpie-robin	*Copsychus saularis*	Insectivore	Resident	LC
104	Oriental Turtle-dove	*Streptopelia orientalis*	Granivore	Resident	LC
105	Oriental White-eye	*Zosterops palpebrosus*	Insectivore	Resident	LC
106	Peregrine Falcon	*Falco peregrinus*	Carnivore	Resident	LC
107	Plumbeous Water-redstart	*Phoenicurus fuliginosus*	Insectivore	Resident	LC
108	Puff-throated Babbler	*Pellorneum ruficeps*	Insectivore	Resident	LC
109	Red-throated Flycatcher	*Ficedula albicilla*	Insectivore	Winter	LC

				visitor	
110	Red-billed Blue Magpie	*Urocissa erythroryncha*	Omnivore	Resident	LC
111	Red-billed Leiothrix	*Leiothrix lutea*	Insectivore	Resident	LC
112	Red-headed Vulture	*Sarcogyps calvus*	Carnivore	Resident	CR
113	Red-vented Bulbul	*Pycnonotus cafer*	Omnivore	Resident	LC
114	Rosy Pipit	*Anthus roseatus*	Insectivore	Winter visitor	LC
115	Rock Dove	*Columba livia*	Granivore	Resident	LC
116	Rose-ringed Parakeet	*Psittacula krameri*	Frugivore	Resident	LC
117	Rufous-gorgeted Flycatcher	*Ficedula strophiata*	Insectivore	Resident	LC
118	Rufous-chinned Laughingthrush	*Garrulax rufogularis*	Insectivore	Resident	LC
119	Rufous-bellied Niltava	*Niltava sundara*	Insectivore	Resident	LC
120	Rufous Woodpecker	*Micropternus brachyurus*	Insectivore	Resident	LC
121	Rufous-throated Partridge	*Arborophila rufogularis*	Omnivore	Resident	LC
122	Rusty-cheeked Scimitar-babbler	*Erythrogenys erythrogenys*	Insectivore	Resident	LC
123	Slaty-headed Parakeet	*Psittacula himalayana*	Frugivore	Resident	LC
124	Slaty-backed Flycatcher	*Ficedula erithacus*	Insectivore	Winter visitor	LC
125	Scaly Thrush	*Zoothera dauma*	Insectivore	Winter visitor	LC
126	Scaly-breasted Cupwing	*Pnoepyga albiventer*	Insectivore	Resident	LC

127	Scaly-breasted Munia	*Lonchura punctulata*	Omnivore	Resident	LC
128	Scarlet Minivet	*Pericrocotus flammeus*	Insectivore	Resident	LC
129	Shikra	*Accipiter badius*	Carnivore	Resident	LC
130	Slender-billed Vulture	*Gyps tenuirostris*	Carnivore	Resident	CR
131	Speckled Piculet	*Picumnus innominatus*	Insectivore	Resident	LC
132	Small Niltava	*Niltava macgrigoriae*	Insectivore	Resident	LC
133	Spiny Babbler	*Acanthoptila nipalensis*	Insectivore	Resident	LC
134	Spotted Forktail	*Enicurus maculatus*	Insectivore	Resident	LC
135	Spotted Owlet	*Athene brama*	Carnivore	Resident	LC
136	Snowy-browed Flycatcher	*Ficedula hyperythra*	Insectivore	Resident	LC
137	Striated Prinia	*Prinia crinigera*	Insectivore	Resident	LC
138	Tickell's Leaf-warbler	*Phylloscopus affinis*	Insectivore	Resident	LC
139	Thick-billed Warbler	*Arundinax aedon*	Insectivore	Winter visitor	LC
140	Velvet-fronted Nuthatch	*Sitta frontalis*	Insectivore	Resident	LC
141	Wallcreeper	*Tichodroma muraria*	Insectivore	Winter visitor	LC
142	Western Koel	*Eudynamys scolopaceus*	Frugivore	Resident	LC
143	Verditer Flycatcher	*Eumyias thalassinus*	Insectivore	Resident	LC
144	White Wagtail	*Motacilla alba*	Insectivore	Winter visitor	LC
145	Wedge-tailed Green-pigeon	*Treron sphenurus*	Frugivore	Resident	LC
146	Western Spotted Dove	*Spilopelia suratensis*	Granivore	Resident	LC
147	White-tailed Nuthatch	*Sitta himalayensis*	Insectivore	Resident	LC

148	White-browed Wagtail	*Motacilla maderaspatensis*	Insectivore	Resident	LC
149	White-bellied Erpornis	*Erpornis zantholeuca*	Insectivore	Resident	LC
150	Western Yellow Wagtail	*Motacilla flava*	Insectivore	Winter visitor	LC
151	White-breasted Kingfisher	*Halcyon smyrnensis*	Carnivore	Resident	LC
152	White-browed Shrike-babbler	*Pteruthius aeralatus*	Insectivore	Resident	LC
153	White-crested Laughingthrush	*Garrulax leucolophus*	Omnivore	Resident	LC
154	White-capped Water-redstart	*Phoenicurus leucocephalus*	Insectivore	Resident	LC
155	White-rumped Munia	*Lonchura striata*	Granivore	Resident	LC
156	White-rumped Vulture	*Gyps bengalensis*	Carnivore	Resident	CR
157	Whistler's Warbler	*Phylloscopus whistleri*	Insectivore	Resident	LC
158	Whiskered Yuhina	*Yuhina flavicollis*	Insectivore	Resident	LC
159	White-throated Laughingthrush	*Garrulax albogularis*	Insectivore	Resident	LC
160	White-throated Fantail	*Rhipidura albicollis*	Insectivore	Resident	LC

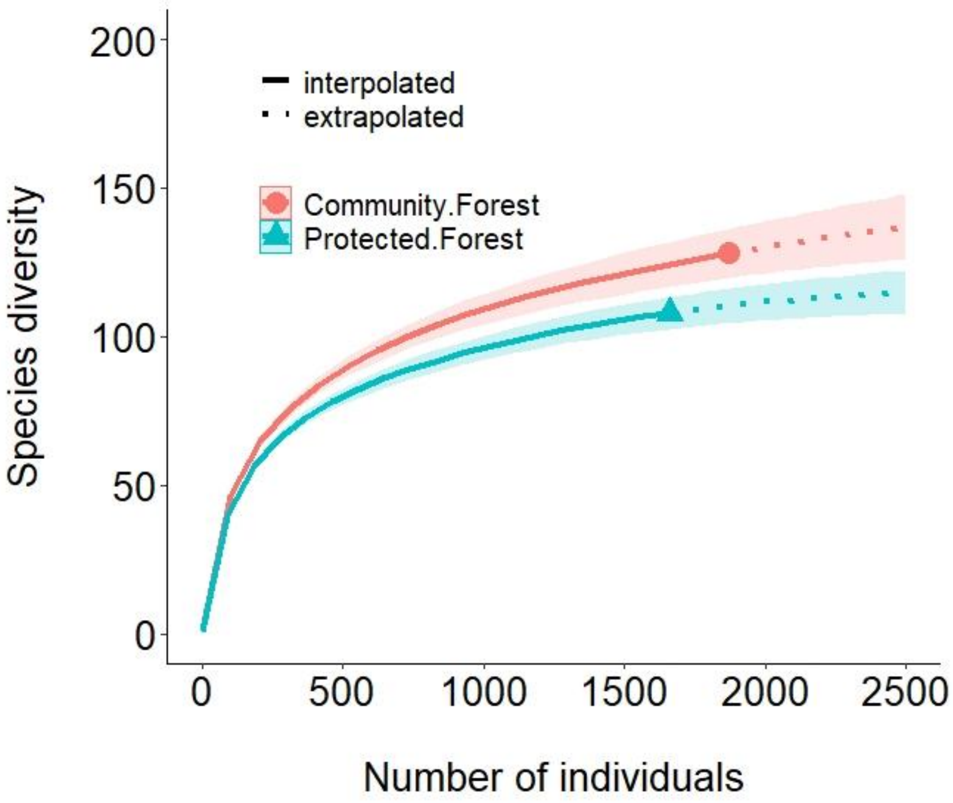

Figure S1. Sample-size-based rarefaction (solid lines) and extrapolation sampling curves with 95% confidence intervals (shaded) representing bird communities in Panchase Protection Forest (protected forest) and Tibrekot Community Forest (community forest).
